# Novel insight of N^6^-methyladenosine modified subtypes in abdominal aortic aneurysm

**DOI:** 10.3389/fgene.2022.1055396

**Published:** 2022-11-22

**Authors:** Kangjie Wang, Qinghui Kan, Yanchen Ye, Jiachong Qiu, Lin Huang, Ridong Wu, Chen Yao

**Affiliations:** ^1^ Division of Vascular Surgery, The First Affiliated Hospital, Sun Yat-Sen University, Guangzhou, China; ^2^ National-Guangdong Joint Engineering Laboratory for Diagnosis and Treatment of Vascular Disease, First Affiliated Hospital, Sun Yat-Sen University, Guangzhou, China; ^3^ Department of Vascular Surgery, The Second Affiliated Hospital of Nanchang University, Nanchang, China

**Keywords:** N^6^-methyladenosine, methylation, abdominal aortic aneurysm, immune infiltration, m6Asore

## Abstract

**Background:** N^6^-methyladenosine (m6A) is the most prevalent non-cap reversible modification present in messenger RNAs and long non-coding RNAs, and its dysregulation has been linked to multiple cardiovascular diseases, including cardiac hypertrophy and atherosclerosis. Although limited studies have suggested that m6A modification contributes to abdominal aortic aneurysm (AAA) development, the full landscape of m6A regulators that mediate modification patterns has not been revealed.

**Methods:** To distinguish the m6A methylation subtypes in AAA patients, an unsupervised clustering method was carried out, based on the mRNA levels of 17 m6A methylation regulators. Differentially expressed genes were identified by comparing clusters. An m6Ascore model was calculated using principal component analysis and structured to assess the m6A methylation patterns of single samples. Subsequently, the relationship between the m6Ascore and immune cells and the hallmark gene set was analyzed. Finally, pairs of circRNA-m6A regulators and m6A regulators-m6A related genes were used to establish a network.

**Results:** We identified three m6A methylation subtypes in the AAA samples. The m6Acluster A and C were characterized as more immunologically activated because of the higher abundance of immune cells than that in m6Acluster B. The m6Acluster B was less enriched in inflammatory pathways and more prevalent in pathways related to extracellular matrix stability. Subsequently, we divided the individual samples into two groups according to the m6Ascore, which suggested that a high m6Ascore predicted more active inflammatory pathways and higher inflammatory cell infiltration. A network consisting of 9 m6A regulators and 37 circRNAs was constructed.

**Conclusion:** This work highlighted that m6A methylation modification was highly correlated with immune infiltration of AAA, which may promote the progression of AAA. We constructed an individualized m6Ascore model to provide evidence for individualized treatments in the future.

## 1 Introduction

Characterized by the segmental and permanent dilatation of the abdominal aorta by over 50% and the consistent weakening of the aortic wall (Thompson et al., 2002), AAA is a common but devastating vascular disease which, although typically asymptomatic, has a mortality rate exceeding 80% after rupture (Golledge and Norman, 2011). At present, open surgical repair and endovascular placement of a stent graft are the mainstays of treatment for AAA, even though only 10% of patients are eligible for surgery (Vandestienne et al., 2021). There are no pharmacological therapies to limit the progression or reduce the risk of rupture (Chaer et al., 2020), and treatment of the latter remains a challenge.

Since the high mortality of ruptured AAA evokes public concern, emerging basic research has focused on the etiopathogenesis to suppress its deterioration. AAA is mainly characterized by the disintegration of the extracellular matrix (ECM), infiltration of inflammatory cells, dysfunction of vascular smooth muscle cells (VSMCs) including phenotypic switch, apoptosis, necroptosis and senescence (Lu et al., 2021), and production of excessive reactive oxygen species (ROS) that cause the destruction of the aortic wall and result in its progressive dilatation (Davis et al., 2015; Golledge, 2019; Gurung et al., 2020). In addition, AAA is an inflammatory condition. Previous studies have confirmed a host of inflammatory cells infiltrating the aortic walls, which demonstrated their significant roles in AAA (Raffort et al., 2017; Yuan et al., 2020). For instance, macrophages severely disequilibrate reparative tissue restoration and destructive tissue remodeling by impairing extracellular matrix remodeling and promoting and resolving inflammatory responses (Murray and Wynn, 2011; Raffort et al., 2017). Neutrophils participate in biological processes in AAA, including oxidative stress, inflammation of adventitia, proteolytic degradation of the tunica media and intraluminal thrombogenesis (Plana et al., 2020). However, a comprehensive profile of inflammatory cells in AAA remains to be elucidated.

Chemical modification on RNA molecules is common in eukaryotic cells, of which m6A modification is the most abundant type (Boccaletto et al., 2018; Wang et al., 2020). The reversible m6A modification can influence the process of RNA maturation and cleavage and involve in its metabolism (Zhang et al., 2020a). Recently, there have been novel insights into the variation of m6A methylation in AAA progression (He et al., 2019). *METTL3*, a writer of m6A modification, is involved in AAA formation by promoting mature *miR-34a* expression, thereby decreasing the expression of *SIRT1* (Zhong et al., 2020). Further studies have revealed that dysregulation of m6A methylation might induce AAA progression (He et al., 2019) or rupture (Fu et al., 2022). Although promising, studies on the underlying mechanism of m6A for potential AAA therapy are lacking.

In this study, we characterized AAA samples into three different classes that revealed distinctive m6A patterns. The immune environment and inherent biological pathways varied in each pattern. Therefore, an individualized m6Ascrore was established to quantify the m6A modification levels in each sample. Given the intrinsic m6A pattern differences, a circular RNA (circRNA) mediating m6A regulators network was constructed for the regulation of m6A modifications. Together, these findings lay the foundation for future immunotherapeutic investigations of AAA.

## 2 Materials and methods

### 2.1 Download of abdominal aortic aneurysm dataset and preprocessing

The gene expression profile of AAA was retrospectively obtained from the Gene Expression Omnibus (GEO) online database. In total, 3 AAA cohorts, GSE98278, GSE47472, and GSE57691, comprising 111 AAA patients and 18 normal donors, were selected for in-depth study. Another cohort, GSE7084, containing 7 AAA patients and 8 normal donors, was retrieved for validation. Additionally, a cohort GSE144431, consisting of four AAA patients and four healthy donors was acquired for differentially expressed circRNA analysis. The batch effect was corrected by the “ComBat” method of the SVA package (Leek et al., 2012). The defined different expressed genes (DEGs) were identified based on the screening criteria of |log2FC| ≥ 1 and adjusted. Significance was defined as *p*-value <0.05.

### 2.2 Sample acquisition

This study was approved by the Institutional Ethics Committee for Clinical Research of the First Affiliated Hospital of Sun Yat-sen University (authorized number: [2020]326). The study conformed to the Declaration of Helsinki. Aneurysmal specimens were collected from 5 patients who underwent open aortic aneurysm repair, and normal aorta samples were obtained from 5 healthy donors without abdominal aortic disease. After divided the tissues into vascular segments, some tissues were fixed with paraformaldehyde (4%) for follow-up immunohistochemistry, and other parts were frozen in liquid nitrogen for follow-up RNA extraction and western blot analysis.

### 2.3 Quantitative real-time polymerase chain reaction

Total RNA from the normal aorta and AAA tissues was extracted using the TRIzol method (Invitrogen, Thermo Fisher Scientific. Co., Ltd.). Next, cDNA was synthesized using an Evo M-MLV Mix Kit (Accurate Biology, AG11728, Hunan, China). Quantitative analyses were performed using qRT-PCR according to the SYBR Green method (Accurate Biology, AG11701, Hunan, China). The relative fold changes of three m6A regulators were calculated using the 2^−ΔΔCT^ method. Primers used in this study are provided in supplementary material (Supplementary Table S1).

### 2.4 Western blotting

After collection, the aorta samples were lysed and the protein content quantified using the BCA protein determination method (Fudebio-tech, FD 2001, China). Proteins were separated by SDS-PAGE electrophoresis. The primary antibodies are listed below: anti-*ALKBH5* (Proteintech, catalog, 16837-1-AP, Wuhan, China), anti-*METTL14* (Proteintech, catalog, 80790-1-AP, Wuhan, China), and anti-*YTHDF1* (Proteintech, catalog, 17479-1-AP, Wuhan, China). *GAPDH* (Proteintech, catalog, 60004-1-Ig, Wuhan, China) was used as an internal reference. Finally, the immunoreactive signal was detected with a chemiluminescent detection substrate (Merck, WBKLS0500, Germany).

### 2.5 Immunohistochemistry staining of m6A methylation regulators

In brief, the sections of vascular segments were heated at 60°C for 1 h, washed in xylene 3 times for 15 min each, then hydrated in 85% and 75% ethanol for 5 min each, followed by 3 washes in PBS. Antigen retrieval procedure was implemented by heating the sections in a microwave oven for 3 min using sodium citrate buffer. The sections were then incubated in the dark with 3% H_2_O_2_ for 25 min and washed with PBS 3 times for 5 min each. The sections were incubated with 3% bovine serum albumin (BSA) to block the tissue evenly for 30 min in a humidified chamber at room temperature. Subsequently, sections were incubated with anti-*ALKBH5* (Proteintech, catalog, 16837-1-AP, Wuhan, China), anti-*METTL14* (Proteintech, catalog, 80790-1-AP, Wuhan, China), and anti-*YTHDF1* (Proteintech, catalog, 17479-1-AP, Wuhan, China) at 4°C overnight. Next, the sections were treated with a secondary immunofluorescence antibody. The sections were stained according to the IHC kit protocol of Servicebio, and hematoxylin was used to visualize nuclei. Finally, the integrated optical density (IOD) values of the sections, which denote the staining intensity, were observed under a microscope (Olympus, Japan) and analyzed by Fiji software.

### 2.6 Consensus clustering expression pattern of 17 m6A regulators

By retrieving the literatures, 17 relevant regulators were identified and analyzed for recognizing diverse m6A modification modules. These 17 m6A regulators included six writers (*WTAP*, *RBM15*, *METTL14*, *CBLL1*, *ZC3H13*, and *METTL3*), two erasers (*FTO* and *ALKBH5*), and nine readers (*YTHDC1*, *YTHDC2*, *LRPPRC*, *HNRNPC*, *HNRNPA2B*1, *YTHDF1*, *YTHDF2*, *YTHDF3,* and *ELAVL1*). Consensus clustering of AAA was conducted using the ConsensusClusterPlus algorithm, and classification stability was ensured by conducting 1,000 repetitions (Wilkerson and Hayes, 2010). Principal component analysis of the transcription profiles was performed using R package, FactoMineR. The mRNA level of these regulators was visualized using the R package, pheatmap.

### 2.7 Estimation of immune characteristics of abdominal aortic aneurysm

To estimate the immune environment in AAA, a single-sample gene set enrichment analysis (ssGSEA) was conducted. The gene sets retrieved from the study by Charoentong, which incorporated multiple human immune cell subtypes (natural killer T cells, macrophages, activated dendritic cells, activated CD8 T cells, and regulatory T cells; Supplementary Table S2) were used for cell marking (Plana et al., 2020; Wang et al., 2020). Enrichment scores were used for individual sample estimation. The correlation between m6A regulators and immune cells was evaluated using Spearman’s correlation analysis.

### 2.8 Gene set variation analysis and functional enrichment analysis

GSVA was performed between m6A modification patterns using the R package GSVA. Conceptually, GSVA is a nonparametric and unsupervised method that evaluates alterations in biological processes or signaling pathway activity in a single sample (Hänzelmann et al., 2013). The gene set of “c2.cp.kegg.v7.5.1.symbols.gmt” and hallmark gene set (h.all.v7.5.1.symbols.gmt) were acquired from the MSigDB database. Moreover, the mRNA expression of immune checkpoints (Supplementary Table S3) and human leukocyte antigen (HLA) molecules (Supplementary Table S4) in each sample captured from the current literature were estimated (Zhao et al., 2021). The activated and repressed pathways were further analyzed using the R package limma (Ritchie et al., 2015). The signaling pathway was considered statistically significant, with an adjusted threshold (*p*-value <0.05). Biological function enrichment for m6A-related DEGs was implemented using the clusterProfiler R package, with a cutoff value of adjusted *p*-value <0.05. The top 30 enriched biological processes are shown as dot plots.

### 2.9 Identification of m6A patterns related gene signature

Based on the expression of 17 m6A molecules, AAA samples were divided into three distinct m6A associated patterns in order to recognize m6Acluster-related genes. DEGs between different modification patterns were identified on the basis of |log2FC| ≥ 0.5 and adjusted (*p*-value <0.001).

The m6Ascore was defined and constructed to quantify the m6A modification level of each patient. The establishment procedures of m6A gene signature were mentioned in brief: Firstly, 625 DEGs were recognized from above different m6Aclusters. Secondly, a consensus clustering method based on the transcript profiles of these DEGs was used to classify the patients into several gene clusters. Next, Z-score transformation of these genes was implemented, followed by PCA analysis. Principal components 1 and 2, which maintained the features that contributed the most to the variance, were chosen to construct the signature scores. Finally, as in previous studies, the following formula was adopted to define the m6Ascore (Sotiriou et al., 2006; Zhang et al., 2020b):

m6Ascore=∑(PC1i+PC2i)

where i is the expression level of m6A methylation pattern-related DEGs.

### 2.10 Characteristics of immune and transcriptome traits in m6Ascore phenotypes

Spearman’s correlation analysis was performed to quantify the correlation between the m6Ascore and immune infiltration, hallmark gene set, HLA molecules, and immune checkpoints. Based on the median of all m6Ascore, the patients were classified into high and low m6Ascore groups. Kruskal–Wallis test was performed to estimate the enrichment score of immune infiltration, HLA molecules, and immune checkpoints between these two groups. The differentially expressed circRNAs were screened out with a threshold of |log2FC| ≥ 2 and *p*-value <0.05. The circRNAs-m6A regulators and m6A regulators-m6A related genes interacting pairs were downloaded from the ENCORI database to further analyze the circRNA-mediated regulatory network in m6A related genes (Li et al., 2014).

### 2.11 Statistical analyses

The Shapiro-Wilk normality test and Bartlett homogeneity test were used to determine the normality and homogeneity of variance, respectively. The Wilcoxon test or Kruskal–Wallis test was used to compare nonparametric variables, while the *t*-test or one-way ANOVA was used to compare parametric variables. Spearman’s correlation analysis was used to calculate correlation coefficients. The effectiveness of the model was confirmed using a receiver operating characteristic curve (ROC). All statistical analyses were two-sided, and *p* < 0.05 was considered statistically significant.

## 3 Results

### 3.1 Transcriptional alterations of 17 m6A methylation regulators in AAA

**FIGURE 1:**
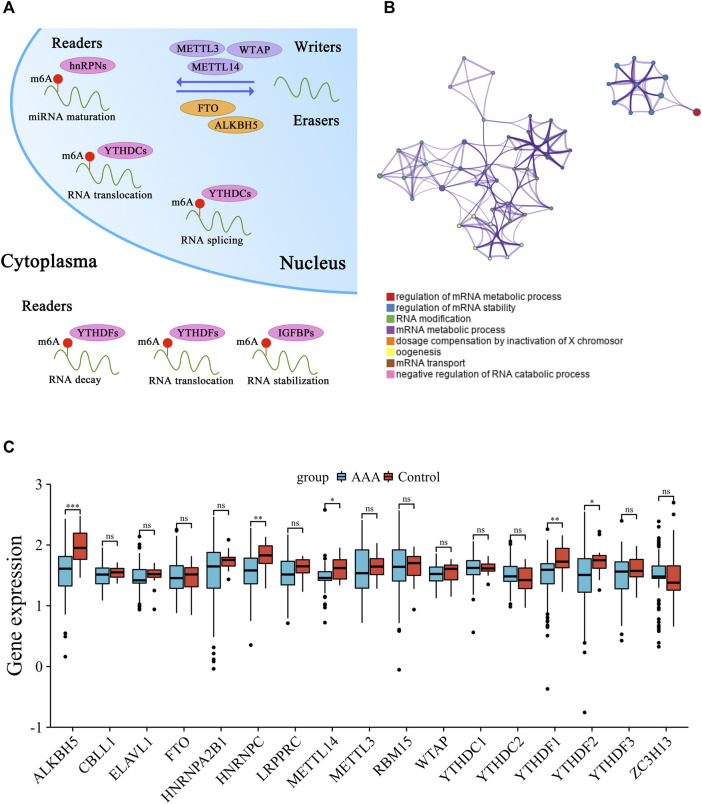
Transcriptional alterations of 17 m6A methylation regulators in AAA. **(A)** The schematic diagram of dynamic and reversible process of m6A RNA methylation. **(B)** The biological process annotations of 17 m6A methylation regulators analyzed by Metascape website tool. **(C)** Boxplot of the discrepant expression of m6A methylation regulators between AAA and control artery samples. AAA: blue; Control: red. (ns: no significance, *p < 0.05, **p < 0.01, ***p < 0.001).

After systematically reviewing published articles related to m6A methylation, a total of 17 m6A regulatory genes in AAA were recognized and selected for further analysis, including 6 writers, 2 erasers, and 9 readers. The mechanism of m6A RNA methylation and the potential molecular functions of m6A regulators for RNA are summarized (Figure 1A). Based on the Metascape web tool, 17 m6A methylation regulators were annotated for involving RNA modification and metabolism (Figure 1B). The different expression of these m6A methylation regulators in normal and AAA samples were compared to verify their transcriptional alterations (Figure 1C). Some m6A methylation regulators (e.g., *ALKBH5*, *HNRNPC*, *METTL14*, *YTHDF1*, and *YTHDF2*) had significantly lower expression in AAA tissues than in normal aorta arteries, suggesting that imbalanced expression of m6A regulators plays a vital role in the formation and progression of AAA.

### 3.2 Clinical features of 17 m6A methylation regulators

**FIGURE 2:**
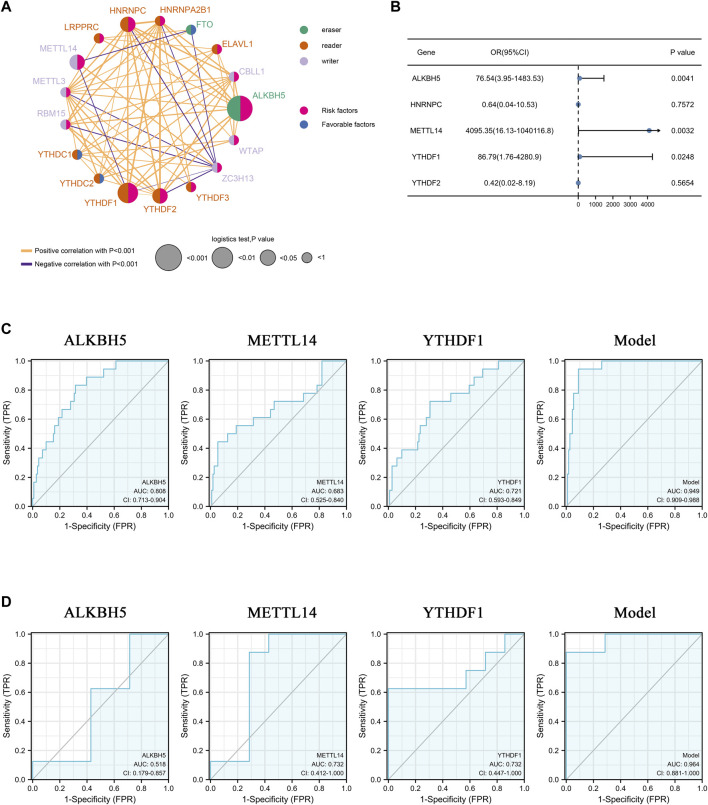
Clinical features of 17 m6A methylation regulators. **(A)** The interaction network among m6A methylation genes. The circle size represents the effect on the prediction and scale by *p*-value. Risk factors: pink; Favorable factors: blue; Erasers: green; Readers: orange; Writers: purple. The orange and purple lines represent positive and negative correlations, respectively (*p* < 0.001). **(B)** Forest plot of multiple logistic regression of 5 m6A methylation regulators. Blue dots represent the odd ratio of each regulator. **(C,D)** ROC curve of m6A regulators in predicting the occurrence of AAA. **(C)**: training set, **(D)** validation set. AUC: area under the curve. CI: 95% confidence interval.

The predictive value of 17 m6A methylation regulators in AAA patients was calculated using the univariate logistic regression model (Supplementary Table S5). The m6A gene regulatory network was employed to depict the comprehensive landscape of m6A methylation regulator connections and interactions, as well as their predictive value in patients with AAA (Figure 2A). 14 regulators (e.g., *HNRNPC*, *METTL14*, *YTHDF1*, *YTHDF2*, *ALKBH5*) were considered as risk factors for AAA, while 3 regulators were favorable factors. These m6A regulators presented significant correlations not only in the same functional category but also among readers, writers, and erasers. The expression of *ZC3H13* (a writer) was inversely correlated with that of *FTO* (an eraser), *HNRNPA2B1*, *HNRNPC*, *LRPPRC*, *YTHDF2*, *YTHDF1* (readers), and *METTL3* and *RBM15* (writers), and the expression level of *METTL14* was inversely correlated with *FTO* and *YTHDF1*. Five regulators were identified as statistically promising risk factors by univariate logistic regression analysis. However, only *ALKBH5*, *METTL14*, and *YTHDF1* were found to be significant risk factors in multiple logistic regression analysis (Figure 2B). Therefore, ROC evaluation was subsequently performed to explore the accuracy of prediction of these three m6A regulators in AAA diagnosis. The results of the training set (Figure 2C) and validation set (Figure 2D) showed that these m6A regulators could predict the occurrence of AAA well, and the regulator combination model greatly improved the predictive power.

### 3.3 Validation of expression of ALKBH5, METLL14, and YTHDF1

**FIGURE 3:**
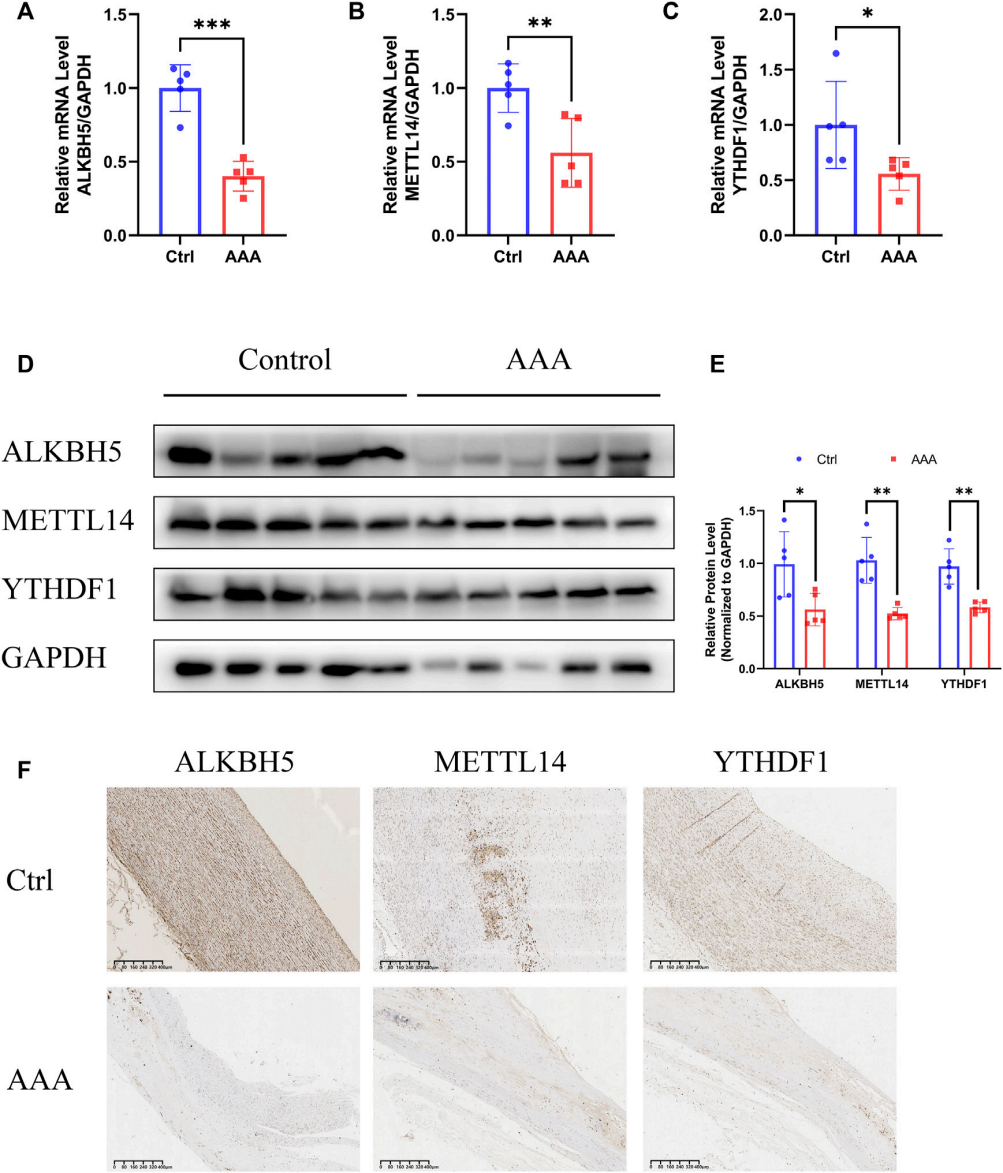
Validation of expression of *ALKBH5*, *METTL14* and *YTHDF1*. **(A–C)** The expression of *ALKBH5*, *METTL14* and *YTHDF1*, detected by qRT-PCR assay. *GAPDH* served as the reference gene. Error bars represent the mean ± SD. **(D,E)** The protein levels of *ALKBH5*, *METTL14* and *YTHDF1*, detected by Western blot assay. **(D)** *GAPDH* served as the loading control. Histogram analysis of fold change of protein levels is shown. **(E)** Error bars represent the mean ± SD. **(F)** The expression of *ALKBH5*, *METTL14* and *YTHDF1* in AAAs and normal aortas, detected by IHC. Scale bar, 400 μm. All experiments are performed in biological triplicate. *p < 0.05, **p < 0.01, ***p < 0.001.

To further verify the reliability of the bioinformatics analysis and ensure the authenticity of the results, a validation cohort comprising five AAA patients and five healthy donors was enrolled. As expected, the mRNA levels of *ALKBH5*, *METTL14*, and *YTHDF1* were notably decreased in the AAA samples (Figures 3A–C). Congruously, the protein levels of the three regulators were also downregulated in AAA tissues (Figures 3D,E). Furthermore, we detected the expression of *ALKBH5*, *METTL14*, and *YTHDF1* in histological sections, which indicated that the distribution of these regulators was relatively low in AAA samples (Figure 3F, Supplementary Figures S1A–C). These results demonstrated that the authentic expression of m6A regulators was consistent with the mining data, based on which further analysis was convincing.

### 3.4 Identification of three m6A methylation patterns based on 17 regulators

**FIGURE 4:**
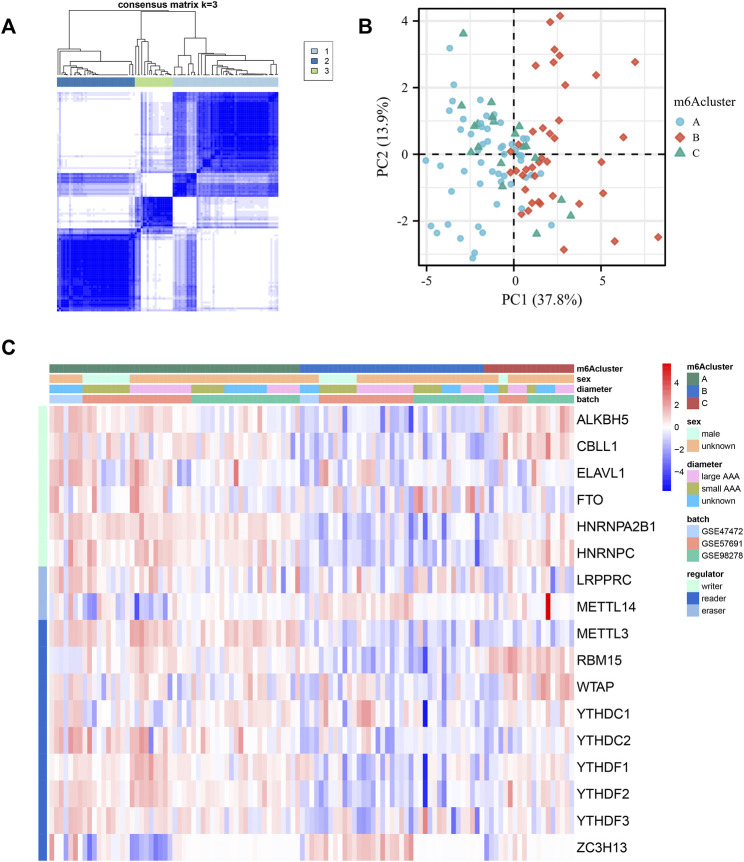
Identification of three m6A methylation patterns based on 17 regulators. **(A)** Consensus clustering of the 17 m6A methylation regulators matrix for k = 3 of 111 patients in the GEO cohort (GSE98278, GSE47472 and GSE57691). **(B)** The principal component analysis of transcriptional profile of the m6A methylation genes of the patients in GEO cohort. **(C)** Unsupervised clustering of the methylation regulators. m6Acluster, sex, AAA diameter, batch, and methylation modification types are used for annotations. Red: high expression. Blue: low expression.

To determine the expression patterns of these 17 m6A regulators, consensus clustering was performed, and three clusters were separated. Three qualitatively different methylation patterns, including 53 cases in m6Acluster_A, 39 cases in m6Acluster_B, and 19 cases in m6Acluster_C, were identified (Figure 4A). Obviously, m6Acluster_B was significantly different from the other two clusters in the transcriptional profile of m6A regulators, according to the PCA analysis (Figure 4B). However, m6Acluster_C is similar to m6Acluster_A. Most methylation regulators, including readers, writers and erasers had dramatically decreased transcriptional levels in m6Acluster_B, followed by m6Acluster_A and m6Acluster_C (Figure 4C).

### 3.5 Three methylation subtypes associated with distinct immune infiltration and molecular backgrounds

**FIGURE 5:**
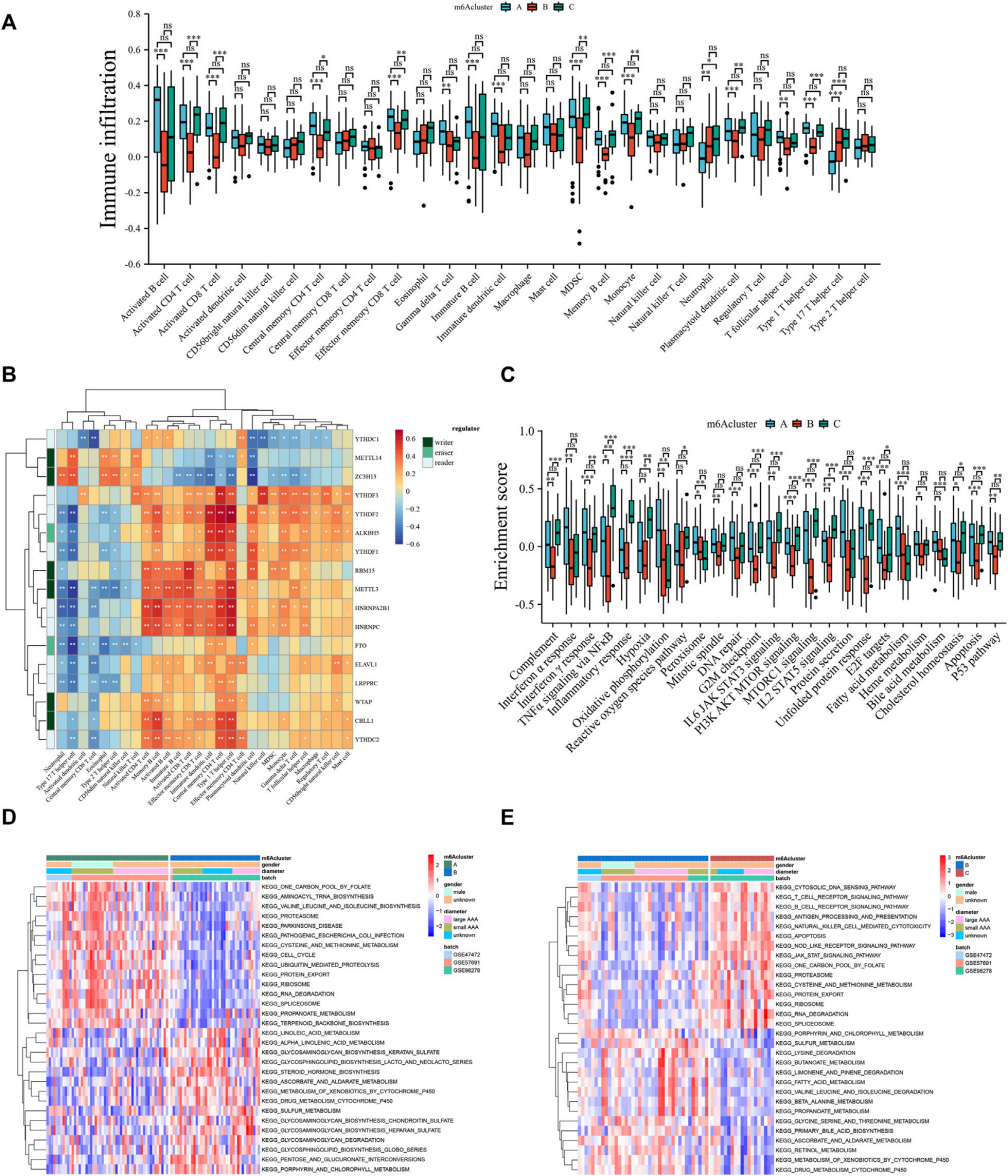
Three methylation subtypes associated with distinct immune infiltration and molecular backgrounds. **(A)** Comparison of the abundance of infiltrating immune cells among three m6Aclusters. **(B)** The Spearman correlation analysis between regulators and immune cells. **(C)** GSVA enrichment analysis among three m6Aclusters based on the hallmark gene sets. **(D,E)** GSVA enrichment analysis among three distinct m6Aclusters based on KEGG pathways. Red: activated pathway. Blue: inhibited pathways. Ns: no significance, *p < 0.05, **p < 0.01, ***p < 0.001.

Some studies have shown significant relevance between immune cell activity and modified methylation (Liu et al., 2021a; Song et al., 2021). Therefore, a comprehensive bioinformatic analysis was performed to reveal the difference in immune activity between methylation subtypes. Based on the analysis of immune cell infiltration among the three different methylation patterns, m6Acluster_B was remarkably different from the other two clusters (Figure 5A). m6Acluster_B had fewer plasmacytoid dendritic cells than m6Acluster_A and m6Acluster_C, suggesting a weak function of active antigen-presenting cells in this group. Additionally, activated CD8^+^ T cells, along with other types of T cells and B cells, were less abundant in m6Acluster_B than in the other two clusters. Known as immune suppressive cells (Tesi, 2019), myeloid-derived suppressor cells (MDSC) were significantly lower in m6Acluster_B, which is contrary to our hypothesis. An intensive correlation between m6A molecules and immune cells was determined using Spearman’s correlation analyses (Figure 5B). For example, a substantial portion of effector T cells and B cells are positively related to the majority of regulators. Notably, there was a negative correlation between type 17 T helper cells and most regulators. To determine the molecular background differences among the three methylation subtypes, hallmark gene sets associated with AAA progression were used for GSVA analysis (Figure 5C). Compared to m6Acluster_A and m6Acluster_C, m6Acluster_B was less enriched in inflammatory response, oxidative stress, mitosis, protein synthesis, fatty acid metabolism, and apoptosis. GSVA enrichment analysis was conducted based on the KEGG pathway to verify the enrichment results further. The m6Acluster_B was more enriched in glycosaminoglycan biosynthesis and degradation related to the composition of the extracellular matrix, but less enriched in amino acid metabolism, protein export, and RNA degradation than m6Acluster_A (Figure 5D). On the other hand, m6Acluster_C was enriched in immune response, protein export, and RNA degradation, but decreased in biometabolism-related pathways (Figure 5E). These results indicate that different methylation patterns are closely related to biological behavior in AAA, and the relatively high activity of methylation regulators may be critical for improving the inflammatory microenvironment in AAA.

### 3.6 Generation of a quantized model for scoring methylation subtypes of individual abdominal aortic aneurysm patients

**FIGURE 6:**
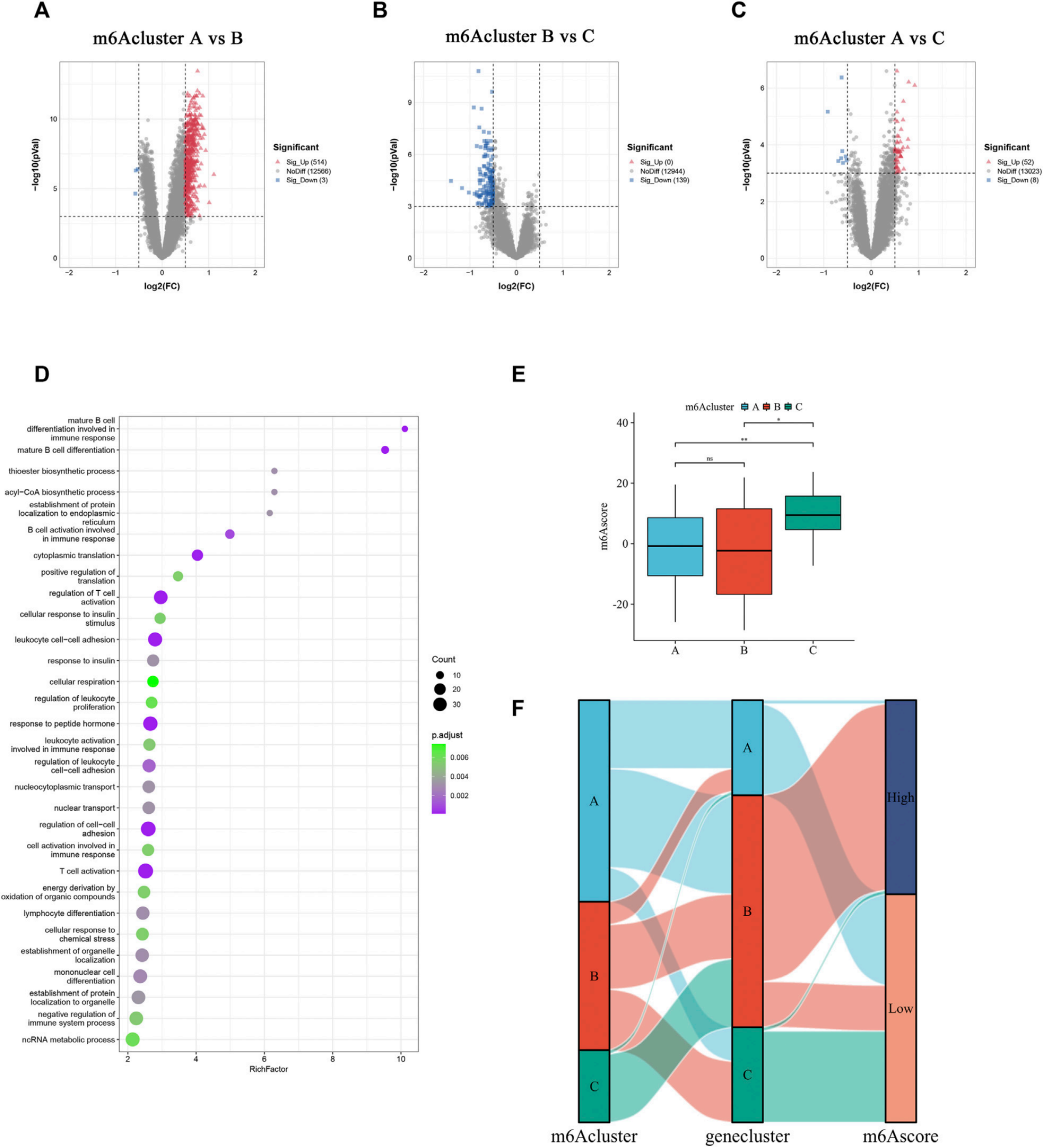
Generation of a quantized model for scoring methylation subtypes of individual AAA patients. **(A–C)** DEGs between three m6Aclusters. The up-regulated and down-regulated DEGs are signed in red and blue. **(D)** The top 30 GO enrichment annotation of DEGs. **(E)** Comparison of the m6Ascore in different m6Aclusters. **(F)** Alluvial diagram shows the alteration in the m6Acluster, geneCluster, and m6Ascore subgroups. Ns: no significance, *p < 0.05, **p < 0.01.

To understand the biological feature differences among the three m6Aclusters comprehensively, 626 DEGs were identified by comparing the three distinct m6Aclusters (Figures 6A–C). Gene Ontology (GO) term enrichment analysis revealed that these DEGs were mainly enriched in immune cell differentiation and activation, nucleocytoplasmic transport, and biosynthetic processes (Figure 6D). According to these DEGs, three clusters termed geneCluster_A, geneCluster_B, and geneCluster_C were separated from each other (Supplementary Figure S2A). Most methylation regulators were markedly upregulated in geneCluster_A (Supplementary Figure S2B). Similarly, the transcriptional profile of geneCluster_A was different from that of cluster B and C (Supplementary Figure S2C). To portray and quantify the methylation pattern of single AAA patients precisely and conveniently, a score model, named m6Ascore, was constructed based on the principal components 1 and 2 of these DEGs. The m6Ascore in m6Acluster_C was remarkably higher than that in m6Acluster_A and m6Acluster_B (Figure 6E). Next, AAA patients were divided into two groups based on the median of m6Ascore in all samples. As a result, samples in the high m6Ascore group were primarily from m6Acluster_A, while the majority of samples from m6Acluster_B and m6Acluster_C were included in the low m6Ascore group (Figure 6F).

### 3.7 Distinction of immune environment and transcriptome characteristics in m6Ascore subgroups

**FIGURE 7:**
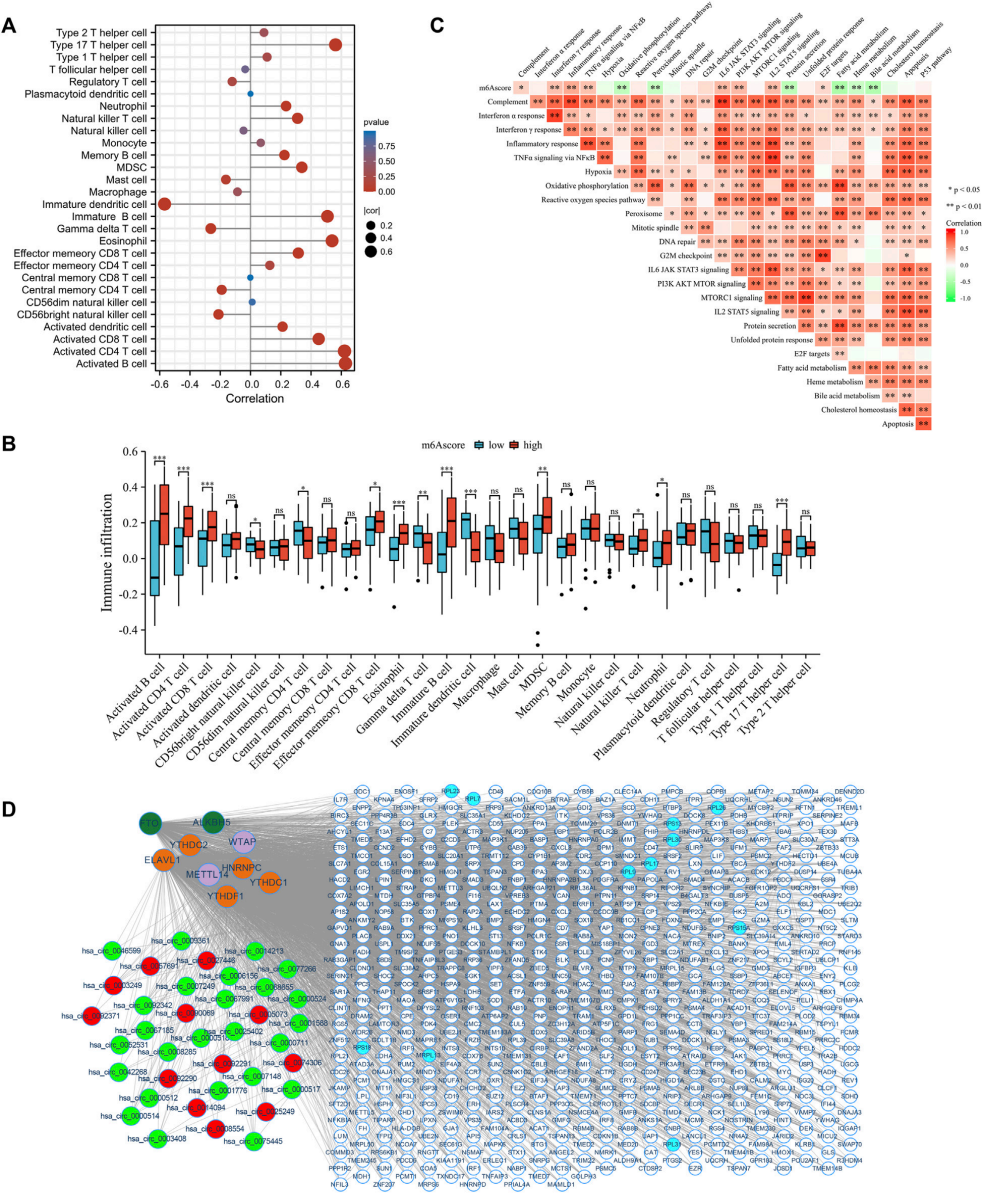
Distinction of immune environment and transcriptome characteristics in m6Ascore phenotypes. **(A)** The correlation between immune cells abundance and m6Ascore in GEO cohort using Spearman analysis. Correlation coefficients are described by circle size and *x*-axis. The color of the circle is scaled by *p*-value. **(B)** The correlation between hallmark gene sets and m6Ascore in GEO cohort using Spearman analysis. Positive and negative correlations are colored with red and green, respectively. **(C)** The relative abundance of immune cells between low and high m6Ascore groups. **(D)** Construction of circRNAs-m6A regulators-mRNA network. Red: upregulated circRNAs. Green: downregulated circRNAs. Atrovirens: erasers. Orange: readers. Purple: writers. White: DEGs. Blue: hub genes. ns: no significance; *p < 0.05; **p < 0.01; ***p < 0.001.

The correlation between immune cells and the m6Ascore was further analyzed to determine the role of m6Ascore-related phenotypes in immune cell infiltration (Figure 7A). The m6Ascore score positively correlated with most T helper and effector T cells. The Kruskal–Wallis test revealed that activated B cells, CD4 T cells, and CD8 T cells were dramatically abundant in the high m6Ascore group (Figure 7B). The correlation between the hallmark gene set and the m6Ascore was also studied to better demonstrate the characteristics of the m6Ascore (Figure 7C). Notably, the m6Ascore was positively correlated with inflammatory response, *STAT3* signaling, and *MTOR* signaling, and negatively correlated with oxidative phosphorylation, fatty acid metabolism, heme metabolism, and bile acid metabolism, indicating that the m6Ascore was involved in immunoregulation. Further, we found patients with higher m6Ascore were in higher immune checkpoint response (Supplementary Figure S3A) and HLA expression (Supplementary Figure S3B). Since individual m6Ascore for patients were calculated based on the m6A related signature, the alteration of the expression profile could change the m6Ascore. Mounting evidence implies that circRNAs play an important role in regulating gene expression by directly binding to proteins (Huang et al., 2020a; Okholm et al., 2020; Zhou et al., 2020) and participating in m6A methylation modifications by interacting with m6A regulators (Chen et al., 2021; Tang and Lv, 2021; Issah et al., 2022). Therefore, the potential circRNAs that might affect the m6A related signature were identified using a cutoff value of |log2FC| ≥ 2 and *p*-value <0.05. In total, 37 were identified as significantly differentially expressed circRNAs (DEcircRNA), including 25 upregulated and 12 downregulated circRNAs. We examined the interaction between DEcircRNAs and m6A regulators based on the circRNAs-m6A regulators-mRNA network, which comprised 37 circRNAs, 9 m6A regulators, and 588 m6A related genes (Figure 7D). The results revealed that most m6A related genes were likely regulated by circRNAs that could interact with m6A regulators.

## 4 Discussion

AAA is a common but life-threatening disease characterized by dilatation of the abdominal aorta (Golledge and Norman, 2011; Vandestienne et al., 2021). There is still no effective non-surgical treatment for this disease, owing to the limited understanding of its pathogenesis. However, inflammation has been suggested to play a central role in its development (Yuan et al., 2020). A host of inflammatory cells, such as macrophages and lymphocytes, infiltrate the aortic walls and produce inflammatory cytokines (e.g., *IL-1β* and *NLRP3*), leading to the loss of structural stability and dilation of the walls (Investigators, 2017; Ma et al., 2022). As the most abundant type of RNA modification in eukaryotic cells, dynamic m6A modification commands a host of cellular biological processes including alternative splicing, mRNA translation, and RNA stability (Yang et al., 2018). It has been reported that aberrant expression of m6A modified proteins or abnormal m6A modified levels could result in disturbed RNA metabolism and influence gene expression that are in close contact with the development of AAA (Li et al., 2021; Peng et al., 2022). However, the association between overall m6A characterization and immune response in AAA progression remains unclear.

Owing to fatal outcomes, numerous biomarkers or models have been explored to predict the development and progression of AAA. A systematic review showed that serum elastin peptides (SEP) and plasmin-antiplasmin (PAP) complexes have the strongest association with AAA, and matrix-degrading metalloproteinase 9 (*MMP9*) and interferon-gamma (*INF-γ*) are promising for use in clinical applications (Urbonavicius et al., 2008). Another large cohort study determined that several novel microRNAs (miRs) presented expression alterations in AAA patients compared to those in normal patients (Wanhainen et al., 2017). Furthermore, an exponential growth model was able to provide useful information about aneurysm size progression using spherical diameter (Akkoyun et al., 2021). However, there are still no relevant predictors that can achieve clinical translation. In the current study, ROC analysis showed that three m6A regulators, *ALKBH5*, *METTL14*, and *YTHDF1*, could predict AAA occurrence. In particular, the model combining these three m6A regulators achieved an area under the curve (AUC) of 0.964, which was much higher than that of previously studied predictors or models. These results highlighted that the imbalanced expression of m6A regulators contributed to the pathogenesis of AAA, and they were able to offer a new perspective and act as a reliable tool to assist in predicting the development of AAA. To our surprise, these three genes in this study, *ALKBH5*, *METTL14*, and *YTHDF1*, showed a trend of down-regulation in AAA. This was most likely due to the different functions of them and polygenic involvement in regulating the methylation level, which indicated that gene expression levels of several m6A regulators alone cannot reflect the degree of m6A methylation modification completely. As a m6A demethylase, *ALKBH5* may lead to an increase in the overall level of RNA methylation in AAA. *METTL14*, a m6A writer protein, promote RNA m6A modification by assist with *METTL3*. However, m6A medication in RNA leads to its splice, transport, degradation and translation that are mediated by the readers (Li et al., 2022a). Analogously, down-regulation of *METTL14* was regarded as risk factor and correlated with poor prognosis (Yang et al., 2020). Despite regulating RNA translation, the role of *YTHDF1* which whether is beneficial or harmful for AAA progression is not clear. Therefore, more experimental evidences are needed to support this result.

At present, it is suggested that inflammation initiates the pathological process of AAA, followed by degradation of the ECM (Lattanzi, 2020). However, some studies have indicated that media ECM degradation caused by various physical and chemical factors aggravates inflammation and immune cell infiltration (Cai et al., 2021). Both inflammation and ECM degradation are early pathological changes in AAA formation (Quintana and Taylor, 2019). In the current study, three distinct m6A expression patterns were identified in the AAA samples. Among them, the m6Acluster_B showed the lowest m6A regulators expression. The m6Acluster _A and _C were characterized by more intensive immune cell infiltration, while the m6Acluster_B was more enriched in pathways related to extracellular matrix stability. These differences between the m6Aclusters may be due to significant pathological and molecular heterogeneity in AAA, such as cellular heterogeneity and imbalanced m6A methylation expression. The three most prominent inflammatory cells involved in the aortic wall of AAA are macrophages, lymphocytes, and mast cells (Yuan et al., 2020). Acting in a pathogenic and reparative role in AAA, macrophages participate in various aspects of the tissue healing response, such as dynamic accommodation of inflammation and extracellular matrix remodeling (Raffort et al., 2017). No differences were detected in the macrophage numbers between the three m6Aclusters in this study. This may be explained by the fact that macrophage heterogeneous subsets existed but could not be further identified. The number of mast cells is positively correlated with AAA size (Swedenborg et al., 2011). Thus, inhibitors of mast cell degranulation, such as tranilast, have been used to slow AAA progression (Tsuruda et al., 2008). However, a similar difference was not found between our three m6Aclusters, which was probably caused by size homogeneity between them. Another study showed that T cells, rather than macrophages, were the main leukocyte subset in AAA, and they were abundant in perivascular adipose tissue (PVAT) (Sagan et al., 2019). These T cells secrete inflammatory factors such as *IFN-γ*, *IL-4* and *IL-5* to promote the expression of MMPs and induce apoptosis of VSMCs (Li et al., 2019). There were significant differences in various T cells among these three m6Aclusters, indicating that m6A methylation regulators may be related to the effect of different T cell profiles. Therefore, it would be intriguing to further investigate whether any m6A methylation regulators might be involved in inhibiting or promoting the inflammation and ECM degradation of AAA by regulating various T cell subsets.

Inflammation, particularly immune infiltration by activated macrophages, plays a vital role in the development and rupture of AAA (Cheuk and Cheng, 2008). Compared with the positive control AAA wall tissue, the expression of *IL-1β*, *IL-6*, *IL-10*, *MIP-2*, neutrophil and macrophage infiltration, and total *MMP9* activity were significantly increased in the ruptured AAA wall (English et al., 2015). Genes with inflammatory and immune functions, including interleukin 8 (*IL-8*), prostaglandin-endoperoxidase synthase 2 (*COX2*), selectin E (*SELE*), and prokineticin 2 (*PROK2*), were confirmed to be overexpressed in aneurysm rupture lesions (Choke et al., 2009). These inflammatory cells and factors, that might be regulated by m6A methylation regulators, could degrade the ECM, leading to AAA rupture. Altering RNA decay, *ALKBH5*-mediated m6A demethylation enhanced the migration capability of neutrophils, resulting in changes in target protein expression, including increased *NLRP12* expression and decreased *PTGER4* expression (Liu et al., 2022). Interacting with *FOXO1*, *METTL14* acts directly on the promoter regions of *ICAM-1* and *VCAM-1* to upregulate their transcription, thereby mediating the inflammatory response of endothelial cells in atherosclerosis (Jian et al., 2020). Recognizing and binding to the m6A methylation site, *YTHDF1* promotes the translation of *SOCS3* mRNA, thus inhibiting the *JAK2/STAT3* pathway and decreasing the secretion of inflammatory factors, leading to anti-inflammatory regulation in *Treponema pallidum* (TP) infection (Li et al., 2022b). Thus, it can be seen that m6A methylation regulators are pivotal in the regulation of inflammation. Based on the results of m6Aclusters, the current study constructed a score model, termed m6Ascore, revealing the role of m6Ascore-related phenotypes in immune cell infiltration in AAA patients. Expectedly, m6Acluster_C had the highest m6Ascore, suggesting that a more active m6A methylation related phenotype contributed to the exacerbation of inflammation in AAA. Thus, the m6Ascore could reflect the degree of immune infiltration and thereby help predict AAA rupture. Nevertheless, more experiments are required to verify the clinical efficacy of the m6Ascore in predicting AAA rupture.

As a type of non-coding RNA, some circRNAs have been confirmed to participate in the development of AAA. For example, *hsa_circ_0087352* was reported to promote the inflammatory response of macrophages in AAA by adsorbing *hsa-miR-149-5p* (Murray and Wynn, 2011). Three other circRNAs, *hsa_circ_0011449, hsa_circ_0081968 and hsa-let-7f-5p*, were found to be correlated with the occurrence of AAA by regulating some other inflammatory cytokines, such as *SOD2* and *CCR7* (Zhang et al., 2021). Recently, the crosstalk between circRNAs and m6A methylation is gradually being revealed. CircRNAs can regulate m6A methylation by altering the expression or influencing the function of m6A writers, erasers, and readers (Wang et al., 2021). For instance, *circRNANOTCH1* competitively binds to *METTL14* against *NOTCH1* mRNA, which attenuates the decline in *NOTHC1* (Shen et al., 2021). Moreover, *circZbtb20* ablated the m6A modification of *Nr4a1* mRNA by recruiting *ALKBH5* (Liu et al., 2021b), whereas *circSTAG1* captured *ALKBH5* and suppressed its translocation into the nucleus (Huang et al., 2020b). The current study identified 37 significantly differentially expressed circRNAs and constructed a circRNAs-m6A regulators-mRNA network consisting of 37 circRNAs, 9 m6A regulators, and 588 m6A related genes. This suggests that circRNAs can modulate and change the m6A pattern of AAA. Therefore, these circRNAs and m6A regulators are expected to become clinically novel predictive biomarkers for AAA.

Although the analysis results suggested that an individualized m6Ascore was helpful for quantifying the degree of immune infiltration and clinical prediction of aneurysm rupture, the GEO database lacks relevant clinical data and cannot be studied further. Therefore, further experimental verification should be performed to uncover regulatory mechanisms and relationships.

## 5 Conclusion

This work highlighted that m6A modification was highly correlated with immune infiltration of AAA, which may promote the progression of AAA. We constructed an individualized m6Ascore to provide evidence and guidelines for individualized treatments in the future.

## Data Availability

The original contributions presented in the study are included in the article/Supplementary Material, further inquiries can be directed to the corresponding authors.

## References

[B1] Akkoyun E. Gharahi H. Kwon S. T. Zambrano B. A. Rao A. Acar A. C. (2021). Defining a master curve of abdominal aortic aneurysm growth and its potential utility of clinical management. Comput. Methods Programs Biomed. 208, 106256. PubMed PMID: 34242864; PubMed Central PMCID: PMCPMC8364512. 10.1016/j.cmpb.2021.106256 34242864 PMC8364512

[B2] Boccaletto P. Machnicka M. A. Purta E. Piatkowski P. Baginski B. Wirecki T. K. (2018). Modomics: A database of RNA modification pathways. 2017 update. Nucleic Acids Res. 46 (D1), D303–D7. PubMed PMID: 29106616. 10.1093/nar/gkx1030 29106616 PMC5753262

[B3] Cai D. Sun C. Zhang G. Que X. Fujise K. Weintraub N. L. (2021). A novel mechanism underlying inflammatory smooth muscle phenotype in abdominal aortic aneurysm. Circ. Res. 129 (10), e202–e214. PubMed PMID: 34551587; PubMed Central PMCID: PMCPMC8575453. 10.1161/CIRCRESAHA.121.319374 34551587 PMC8575453

[B4] Chaer R. A. Abularrage C. J. Coleman D. M. Eslami M. H. Kashyap V. S. Rockman C. (2020). The Society for Vascular Surgery clinical practice guidelines on the management of visceral aneurysms. J. Vasc. Surg. 72 (1S), 3S–39S. PubMed PMID: 32201007. 10.1016/j.jvs.2020.01.039 32201007

[B5] Chen N. Tang J. Su Q. Chou W. C. Zheng F. Guo Z. (2021). Paraquat-induced oxidative stress regulates N6-methyladenosine (m(6)A) modification of circular RNAs. Environ. Pollut. 290, 117816. PubMed PMID: 34425375. 10.1016/j.envpol.2021.117816 34425375

[B6] Cheuk B. L. Cheng S. W. (2008). Can local secretion of prostaglandin E2, thromboxane B2, and interleukin-6 play a role in ruptured abdominal aortic aneurysm? World J. Surg. 32 (1), 55–61. PubMed PMID: 17992560. 10.1007/s00268-007-9279-9 17992560

[B7] Choke E. Cockerill G. W. Laing K. Dawson J. Wilson W. R. Loftus I. M. (2009). Whole genome-expression profiling reveals a role for immune and inflammatory response in abdominal aortic aneurysm rupture. Eur. J. Vasc. Endovasc. Surg. 37 (3), 305–310. PubMed PMID: 19111481. 10.1016/j.ejvs.2008.11.017 19111481

[B8] Davis F. M. Rateri D. L. Daugherty A. (2015). Abdominal aortic aneurysm: Novel mechanisms and therapies. Curr. Opin. Cardiol. 30 (6), 566–573. PubMed PMID: 26352243. 10.1097/HCO.0000000000000216 26352243 PMC4624089

[B9] English S. J. Piert M. R. Diaz J. A. Gordon D. Ghosh A. D'Alecy L. G. (2015). Increased 18F-FDG uptake is predictive of rupture in a novel rat abdominal aortic aneurysm rupture model. Ann. Surg. 261 (2), 395–404. PubMed PMID: 24651130; PubMed Central PMCID: PMCPMC4662083. 10.1097/SLA.0000000000000602 24651130 PMC4662083

[B10] Fu C. Feng L. Zhang J. Sun D. (2022). Bioinformatic analyses of the role of m6A RNA methylation regulators in abdominal aortic aneurysm. Ann. Transl. Med. 10 (10), 547. PubMed PMID: 35722410. 10.21037/atm-22-1891 35722410 PMC9201186

[B11] Golledge J. (2019). Abdominal aortic aneurysm: Update on pathogenesis and medical treatments. Nat. Rev. Cardiol. 16 (4), 225–242. PubMed PMID: 30443031. 10.1038/s41569-018-0114-9 30443031

[B12] Golledge J. Norman P. E. (2011). Current status of medical management for abdominal aortic aneurysm. Atherosclerosis 217 (1), 57–63. PubMed PMID: 21596379. 10.1016/j.atherosclerosis.2011.03.006 21596379

[B13] Gurung R. Choong A. M. Woo C. C. Foo R. Sorokin V. (2020). Genetic and epigenetic mechanisms underlying vascular smooth muscle cell phenotypic modulation in abdominal aortic aneurysm. Int. J. Mol. Sci. 21 (17), E6334. PubMed PMID: 32878347. 10.3390/ijms21176334 PMC7504666 32878347

[B14] Hänzelmann S. Castelo R. Guinney J. (2013). Gsva: Gene set variation analysis for microarray and RNA-seq data. BMC Bioinforma. 14, 7. PubMed PMID: 23323831. 10.1186/1471-2105-14-7 PMC3618321 23323831

[B15] He Y. Xing J. Wang S. Xin S. Han Y. Zhang J. (2019). Increased m6A methylation level is associated with the progression of human abdominal aortic aneurysm. Ann. Transl. Med. 7 (24), 797. PubMed PMID: 32042813. 10.21037/atm.2019.12.65 32042813 PMC6989874

[B16] Huang A. Zheng H. Wu Z. Chen M. Huang Y. (2020). Circular RNA-protein interactions: Functions, mechanisms, and identification. Theranostics 10 (8), 3503–3517. PubMed PMID: 32206104; PubMed Central PMCID: PMCPMC7069073. 10.7150/thno.42174 32206104 PMC7069073

[B17] Huang R. Zhang Y. Bai Y. Han B. Ju M. Chen B. (2020). N(6)-Methyladenosine modification of fatty acid amide hydrolase messenger RNA in circular RNA STAG1-regulated astrocyte dysfunction and depressive-like behaviors. Biol. Psychiatry 88 (5), 392–404. PubMed PMID: 32387133. 10.1016/j.biopsych.2020.02.018 32387133

[B18] Investigators M. R. S. (2017). Aortic wall inflammation predicts abdominal aortic aneurysm expansion, rupture, and need for surgical repair. Circulation 136 (9), 787–797. PubMed PMID: 28720724; PubMed Central PMCID: PMCPMC5571881. 10.1161/CIRCULATIONAHA.117.028433 28720724 PMC5571881

[B19] Issah M. A. Wu D. Zhang F. Zheng W. Liu Y. Chen R. (2022). Expression profiling of N6-methyladenosine modified circRNAs in acute myeloid leukemia. Biochem. Biophys. Res. Commun. 601, 137–145. 10.1016/j.bbrc.2022.02.087 35247767

[B20] Jian D. Wang Y. Jian L. Tang H. Rao L. Chen K. (2020). METTL14 aggravates endothelial inflammation and atherosclerosis by increasing FOXO1 N6-methyladeosine modifications. Theranostics 10 (20), 8939–8956. PubMed PMID: 32802173; PubMed Central PMCID: PMCPMC7415798. 10.7150/thno.45178 32802173 PMC7415798

[B21] Lattanzi S. (2020). Abdominal aortic aneurysms: Pathophysiology and clinical issues. J. Intern. Med. 288 (3), 376–378. PubMed PMID: 32301175. 10.1111/joim.13060 32301175

[B22] Leek J. T. Johnson W. E. Parker H. S. Jaffe A. E. Storey J. D. (2012). The sva package for removing batch effects and other unwanted variation in high-throughput experiments. Bioinformatics 28 (6), 882–883. PubMed PMID: 22257669. 10.1093/bioinformatics/bts034 22257669 PMC3307112

[B23] Li J. Xia N. Wen S. Li D. Lu Y. Gu M. (2019). IL (Interleukin)-33 suppresses abdominal aortic aneurysm by enhancing regulatory T-cell expansion and activity. Arterioscler. Thromb. Vasc. Biol. 39 (3), 446–458. PubMed PMID: 30651000; PubMed Central PMCID: PMCPMC6393188. 10.1161/ATVBAHA.118.312023 30651000 PMC6393188

[B24] Li J-H. Liu S. Zhou H. Qu L-H. Yang J-H. (2014). starBase v2.0: decoding miRNA-ceRNA, miRNA-ncRNA and protein-RNA interaction networks from large-scale CLIP-Seq data. Nucleic Acids Res. 42, D92–D97. PubMed PMID: 24297251. 10.1093/nar/gkt1248 24297251 PMC3964941

[B25] Li T. Wang T. Jing J. Sun L. (2021). Expression pattern and clinical value of key m6A RNA modification regulators in abdominal aortic aneurysm. J. Inflamm. Res. 14, 4245–4258. PubMed PMID: 34511965. 10.2147/JIR.S327152 34511965 PMC8412829

[B26] Li X. Ma S. Deng Y. Yi P. Yu J. (2022). Targeting the RNA m(6)A modification for cancer immunotherapy. Mol. Cancer 21 (1), 76. PubMed PMID: 35296338; PubMed Central PMCID: PMCPMC8924732. 10.1186/s12943-022-01558-0 35296338 PMC8924732

[B27] Li Z. Teng M. Jiang Y. Zhang L. Luo X. Liao Y. (2022). YTHDF1 negatively regulates Treponema pallidum-induced inflammation in THP-1 macrophages by promoting SOCS3 translation in an m6A-dependent manner. Front. Immunol. 13, 857727. PubMed PMID: 35444649; PubMed Central PMCID: PMCPMC9013966. 10.3389/fimmu.2022.857727 35444649 PMC9013966

[B28] Liu B. Liu N. Zhu X. Yang L. Ye B. Li H. (2021). Circular RNA circZbtb20 maintains ILC3 homeostasis and function via Alkbh5-dependent m^6^A demethylation of Nr4a1 mRNA. Cell. Mol. Immunol. 18 (6), 1412–1424. PubMed PMID: 33911218. 10.1038/s41423-021-00680-1 33911218 PMC8166869

[B29] Liu X. S. Zhou L. M. Yuan L. L. Gao Y. Kui X. Y. Liu X. Y. (2021). NPM1 is a prognostic biomarker involved in immune infiltration of lung adenocarcinoma and associated with m6A modification and glycolysis. Front. Immunol. 12, 724741. PubMed PMID: 34335635; PubMed Central PMCID: PMCPMC8324208. 10.3389/fimmu.2021.724741 34335635 PMC8324208

[B30] Liu Y. Song R. Zhao L. Lu Z. Li Y. Zhan X. (2022). m6 A demethylase ALKBH5 is required for antibacterial innate defense by intrinsic motivation of neutrophil migration. Signal Transduct. Target. Ther. 7 (1), 194. PubMed PMID: 35764614; PubMed Central PMCID: PMCPMC9240034. 10.1038/s41392-022-01020-z 35764614 PMC9240034

[B31] Lu H. Du W. Ren L. Hamblin M. H. Becker R. C. Chen Y. E. (2021). Vascular smooth muscle cells in aortic aneurysm: From Genetics to mechanisms. J. Am. Heart Assoc. 10 (24), e023601. PubMed PMID: 34796717; PubMed Central PMCID: PMCPMC9075263. 10.1161/jaha.121.023601 34796717 PMC9075263

[B32] Ma X. Xu J. Lu Q. Feng X. Liu J. Cui C. (2022). Hsa_circ_0087352 promotes the inflammatory response of macrophages in abdominal aortic aneurysm by adsorbing hsa-miR-149-5p. Int. Immunopharmacol. 107, 108691. PubMed PMID: 35286916. 10.1016/j.intimp.2022.108691 35286916

[B33] Murray P. J. Wynn T. A. (2011). Protective and pathogenic functions of macrophage subsets. Nat. Rev. Immunol. 11 (11), 723–737. PubMed PMID: 21997792; PubMed Central PMCID: PMCPMC3422549. 10.1038/nri3073 21997792 PMC3422549

[B34] Okholm T. L. H. Sathe S. Park S. S. Kamstrup A. B. Rasmussen A. M. Shankar A. (2020). Transcriptome-wide profiles of circular RNA and RNA-binding protein interactions reveal effects on circular RNA biogenesis and cancer pathway expression. Genome Med. 12 (1), 112. PubMed PMID: 33287884; PubMed Central PMCID: PMCPMC7722315. 10.1186/s13073-020-00812-8 33287884 PMC7722315

[B35] Peng L. Long T. Li F. Xie Q. (2022). Emerging role of m(6) A modification in cardiovascular diseases. Cell. Biol. Int. 46 (5), 711–722. PubMed PMID: 35114043. 10.1002/cbin.11773 35114043

[B36] Plana E. Oto J. Medina P. Fernandez-Pardo A. Miralles M. (2020). Novel contributions of neutrophils in the pathogenesis of abdominal aortic aneurysm, the role of neutrophil extracellular traps: A systematic review. Thromb. Res. 194, 200–208. PubMed PMID: 32788119. 10.1016/j.thromres.2020.07.039 32788119

[B37] Quintana R. A. Taylor W. R. (2019). Cellular mechanisms of aortic aneurysm formation. Circ. Res. 124 (4), 607–618. PubMed PMID: 30763207; PubMed Central PMCID: PMCPMC6383789. 10.1161/CIRCRESAHA.118.313187 30763207 PMC6383789

[B38] Raffort J. Lareyre F. Clement M. Hassen-Khodja R. Chinetti G. Mallat Z. (2017). Monocytes and macrophages in abdominal aortic aneurysm. Nat. Rev. Cardiol. 14 (8), 457–471. PubMed PMID: 28406184. 10.1038/nrcardio.2017.52 28406184

[B39] Ritchie M. E. Phipson B. Wu D. Hu Y. Law C. W. Shi W. (2015). Limma powers differential expression analyses for RNA-sequencing and microarray studies. Nucleic Acids Res. 43 (7), e47. PubMed PMID: 25605792. 10.1093/nar/gkv007 25605792 PMC4402510

[B40] Sagan A. Mikolajczyk T. P. Mrowiecki W. MacRitchie N. Daly K. Meldrum A. (2019). T cells are dominant population in human abdominal aortic aneurysms and their infiltration in the perivascular tissue correlates with disease severity. Front. Immunol. 10, 1979. PubMed PMID: 31552015; PubMed Central PMCID: PMCPMC6736986. 10.3389/fimmu.2019.01979 31552015 PMC6736986

[B41] Shen Y. Li C. Zhou L. Huang J. A. (2021). G protein-coupled oestrogen receptor promotes cell growth of non-small cell lung cancer cells via YAP1/QKI/circNOTCH1/m6A methylated NOTCH1 signalling. J. Cell. Mol. Med. 25 (1), 284–296. PubMed PMID: 33237585; PubMed Central PMCID: PMCPMC7810948. 10.1111/jcmm.15997 33237585 PMC7810948

[B42] Song H. Song J. Cheng M. Zheng M. Wang T. Tian S. (2021). METTL3-mediated m6A RNA methylation promotes the anti-tumour immunity of natural killer cells. Nat. Commun. 12 (1), 5522. 10.1038/s41467-021-25803-0 34535671 PMC8448775

[B43] Sotiriou C. Wirapati P. Loi S. Harris A. Fox S. Smeds J. (2006). Gene expression profiling in breast cancer: Understanding the molecular basis of histologic grade to improve prognosis. J. Natl. Cancer Inst. 98 (4), 262–272. PubMed PMID: 16478745. 10.1093/jnci/djj052 16478745

[B44] Swedenborg J. Mayranpaa M. I. Kovanen P. T. (2011). Mast cells: Important players in the orchestrated pathogenesis of abdominal aortic aneurysms. Arterioscler. Thromb. Vasc. Biol. 31 (4), 734–740. PubMed PMID: 21205988. 10.1161/ATVBAHA.110.213157 21205988

[B45] Tang M. Lv Y. (2021). The role of *N* ^ *6* ^ -methyladenosine modified circular RNA in pathophysiological processes. Int. J. Biol. Sci. 17 (9), 2262–2277. PubMed PMID: 34239354; PubMed Central PMCID: PMCPMC8241720. 10.7150/ijbs.60131 34239354 PMC8241720

[B46] Tesi R. J. (2019). MDSC; the most important cell you have never heard of. Trends Pharmacol. Sci. 40 (1), 4–7. PubMed PMID: 30527590. 10.1016/j.tips.2018.10.008 30527590

[B47] Thompson R. W. Geraghty P. J. Lee J. K. (2002). Abdominal aortic aneurysms: Basic mechanisms and clinical implications. Curr. Probl. Surg. 39 (2), 110–230. PubMed PMID: 11884965. 10.1067/msg.2002.121421 11884965

[B48] Tsuruda T. Kato J. Hatakeyama K. Kojima K. Yano M. Yano Y. (2008). Adventitial mast cells contribute to pathogenesis in the progression of abdominal aortic aneurysm. Circ. Res. 102 (11), 1368–1377. PubMed PMID: 18451339. 10.1161/CIRCRESAHA.108.173682 18451339

[B49] Urbonavicius S. Urbonaviciene G. Honore B. Henneberg E. W. Vorum H. Lindholt J. S. (2008). Potential circulating biomarkers for abdominal aortic aneurysm expansion and rupture–a systematic review. Eur. J. Vasc. Endovasc. Surg. 36 (3), 273–280. PubMed PMID: 18639476. 10.1016/j.ejvs.2008.05.009 18639476

[B50] Vandestienne M. Zhang Y. Santos-Zas I. Al-Rifai R. Joffre J. Giraud A. (2021). TREM-1 orchestrates angiotensin II-induced monocyte trafficking and promotes experimental abdominal aortic aneurysm. J. Clin. Invest. 131 (2), 142468. PubMed PMID: 33258804. 10.1172/JCI142468 33258804 PMC7810476

[B51] Wang X. Ma R. Zhang X. Cui L. Ding Y. Shi W. (2021). Crosstalk between N6-methyladenosine modification and circular RNAs: Current understanding and future directions. Mol. Cancer 20 (1), 121. PubMed PMID: 34560891; PubMed Central PMCID: PMCPMC8461955. 10.1186/s12943-021-01415-6 34560891 PMC8461955

[B52] Wang X. Wu R. Liu Y. Zhao Y. Bi Z. Yao Y. (2020). mA m6A mRNA methylation controls autophagy and adipogenesis by targeting Atg5 and Atg7. Autophagy 16 (7), 1221–1235. PubMed PMID: 31451060. 10.1080/15548627.2019.1659617 31451060 PMC7469583

[B53] Wanhainen A. Mani K. Vorkapic E. De Basso R. Bjorck M. Lanne T. (2017). Screening of circulating microRNA biomarkers for prevalence of abdominal aortic aneurysm and aneurysm growth. Atherosclerosis 256, 82–88. PubMed PMID: 27993388. 10.1016/j.atherosclerosis.2016.11.007 27993388

[B54] Wilkerson M. D. Hayes D. N. (2010). ConsensusClusterPlus: A class discovery tool with confidence assessments and item tracking. Bioinformatics 26 (12), 1572–1573. PubMed PMID: 20427518. 10.1093/bioinformatics/btq170 20427518 PMC2881355

[B55] Yang X. Zhang S. He C. Xue P. Zhang L. He Z. (2020). METTL14 suppresses proliferation and metastasis of colorectal cancer by down-regulating oncogenic long non-coding RNA XIST. Mol. Cancer 19 (1), 46. PubMed PMID: 32111213; PubMed Central PMCID: PMCPMC7047419. 10.1186/s12943-020-1146-4 32111213 PMC7047419

[B56] Yang Y. Hsu P. J. Chen Y. S. Yang Y. G. (2018). Dynamic transcriptomic m(6)A decoration: Writers, erasers, readers and functions in RNA metabolism. Cell. Res. 28 (6), 616–624. PubMed PMID: 29789545; PubMed Central PMCID: PMCPMC5993786. 10.1038/s41422-018-0040-8 29789545 PMC5993786

[B57] Yuan Z. Lu Y. Wei J. Wu J. Yang J. Cai Z. (2020). Abdominal aortic aneurysm: Roles of inflammatory cells. Front. Immunol. 11, 609161. PubMed PMID: 33613530; PubMed Central PMCID: PMCPMC7886696. 10.3389/fimmu.2020.609161 33613530 PMC7886696

[B58] Zhang B. Wu Q. Li B. Wang D. Wang L. Zhou Y. L. (2020). m6A regulator-mediated methylation modification patterns and tumor microenvironment infiltration characterization in gastric cancer. Mol. Cancer 19 (1), 53. PubMed PMID: 32164750. 10.1186/s12943-020-01170-0 32164750 PMC7066851

[B59] Zhang H. Bian C. Tu S. Yin F. Guo P. Zhang J. (2021). Construction of the circRNA-miRNA-mRNA regulatory network of an abdominal aortic aneurysm to explore its potential pathogenesis. Dis. Markers 2021, 9916881. PubMed PMID: 34777635; PubMed Central PMCID: PMCPMC8589483 publication of this paper. 10.1155/2021/9916881 34777635 PMC8589483

[B60] Zhang L. Hou C. Chen C. Guo Y. Yuan W. Yin D. (2020). The role of N6-methyladenosine (m6A) modification in the regulation of circRNAs. Mol. Cancer 19 (1), 105. PubMed PMID: 32522202. 10.1186/s12943-020-01224-3 32522202 PMC7285594

[B61] Zhao L. Lv F. Zheng Y. Yan L. Cao X. (2021). Characterization of an aging-based diagnostic gene signature and molecular subtypes with diverse immune infiltrations in atherosclerosis. Front. Mol. Biosci. 8, 792540. PubMed PMID: 35096968. 10.3389/fmolb.2021.792540 35096968 PMC8792769

[B62] Zhong L. He X. Song H. Sun Y. Chen G. Si X. (2020). METTL3 induces AAA development and progression by modulating N6-methyladenosine-dependent primary miR34a processing. Mol. Ther. Nucleic Acids 21, 394–411. PubMed PMID: 32650237; PubMed Central PMCID: PMCPMC7347714. 10.1016/j.omtn.2020.06.005 32650237 PMC7347714

[B63] Zhou W. Y. Cai Z. R. Liu J. Wang D. S. Ju H. Q. Xu R. H. (2020). Circular RNA: Metabolism, functions and interactions with proteins. Mol. Cancer 19 (1), 172. PubMed PMID: 33317550; PubMed Central PMCID: PMCPMC7734744. 10.1186/s12943-020-01286-3 33317550 PMC7734744

