# Supplementary material for: Novel insight of N6-methyladenosine modified subtypes in abdominal aortic aneurysm
Source: Front Genet. 2022 Nov 22;13:1055396. doi: 10.3389/fgene.2022.1055396 (PMC9723249; doi:10.3389/fgene.2022.1055396)
Supplement: Supplementary file 2 [file Table1.DOCX]

Supplementary Material

# Supplementary Figures
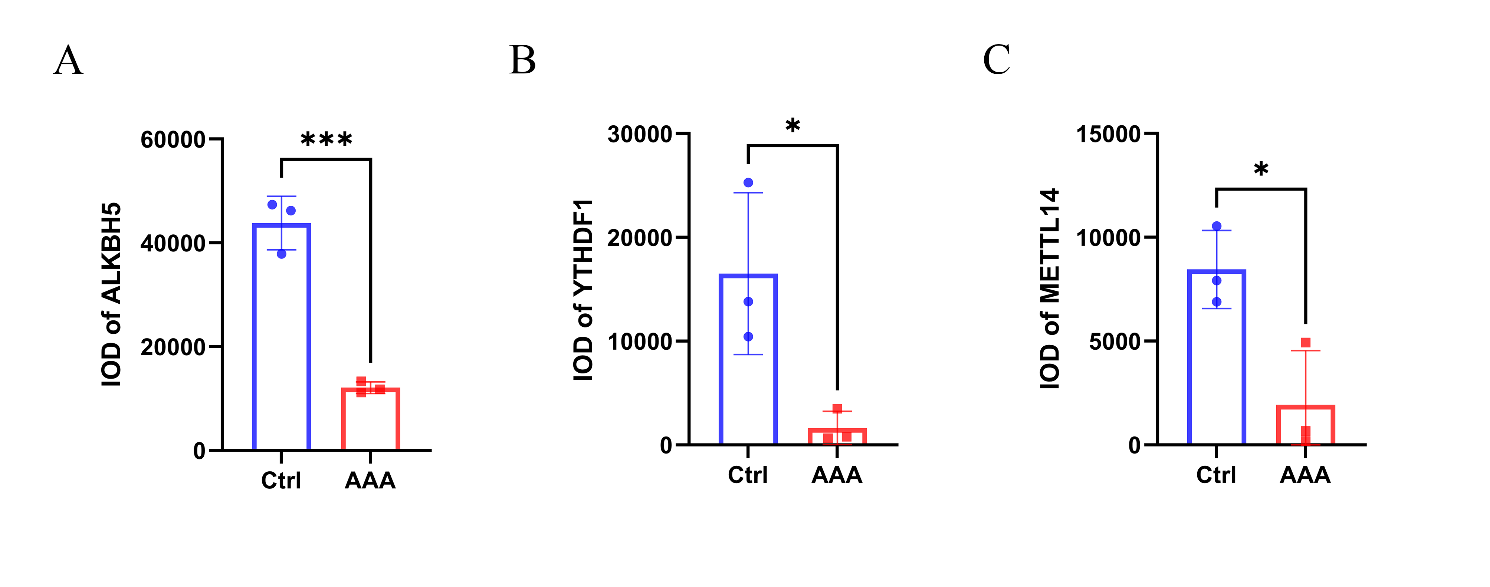
Supplementary Figure 1. Three m^6^A regulators markedly decreased in AAA group compared with control arteries group. IOD, intensity optical density. *, p < 0.05; ***, p < 0.001.


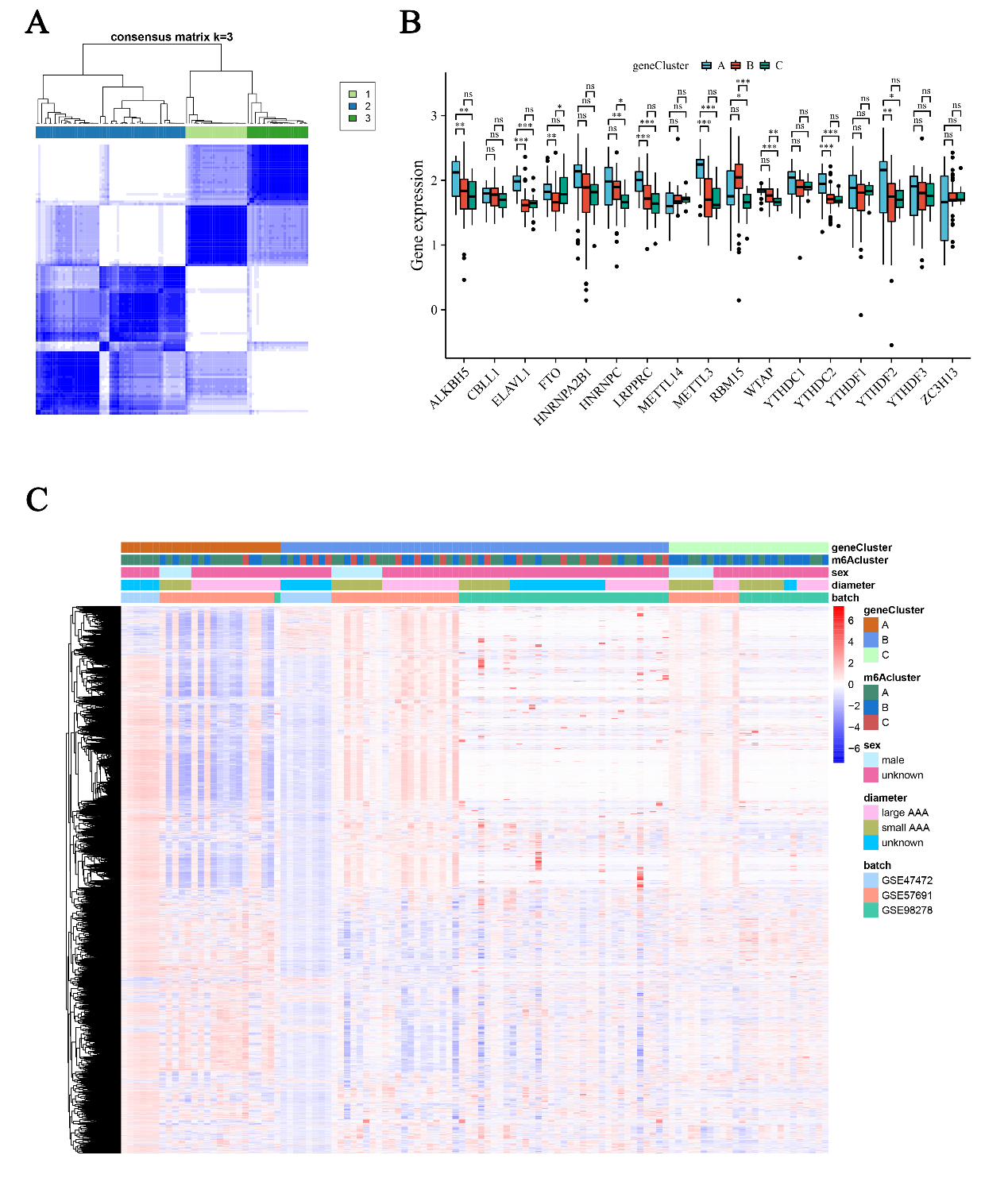
**Supplementary Figure 2.**Identification of three genecluster based on m^6^A-related DEGs. (A) Consensus clustering of the 625 m^6^A-related DEGs matrix for k = 3 of 111 patients in the GEO cohort (GSE99278, GSE47472 and GSE57691). (B) The expression of 17 m^6^A regulators in these geneclusters. (C) Unsupervised clustering of the transcript profile. Genecluster, m^6^Acluster, sex, AAA diameter and batch were used for annotations. Red represents high expression, and blue represents low expression. ns, no significance; *, p < 0.05; **, p < 0.01; ***, p < 0.001.


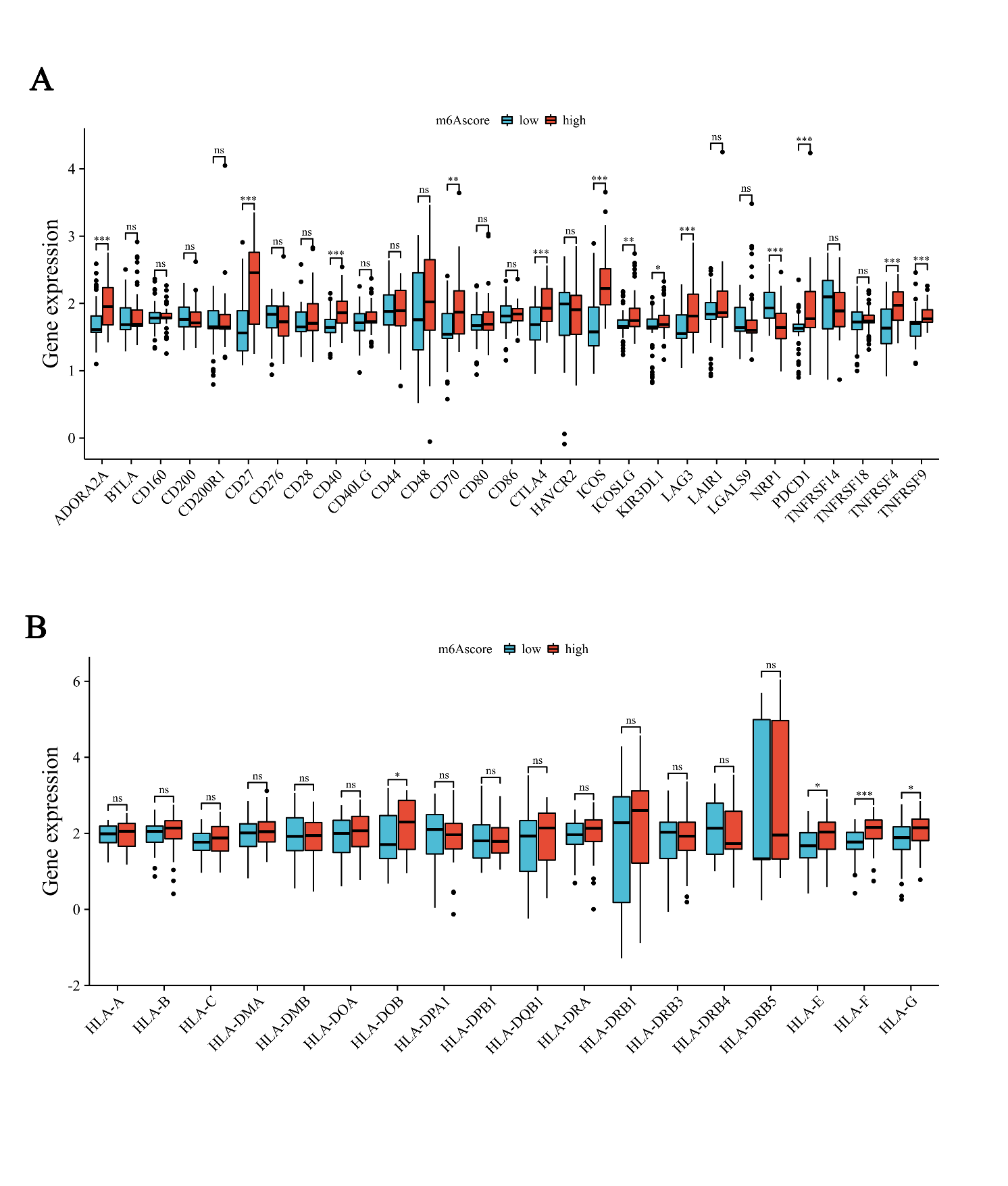


**Supplementary Figure 3.**The immune characteristics between m6Ascore high and low subgroups (A) The expression of immune checkpoint related genes between two groups. (B) The expression of HLA molecules between two groups. ns, no significance; *, p < 0.05; **, p < 0.01; ***, p < 0.001.
